# Editorial: Tumor metabolic microenvironment: one man’s meat is another man’s poison

**DOI:** 10.3389/fimmu.2024.1422624

**Published:** 2024-05-13

**Authors:** Jing Xu, Haitao Wang, Xiao Liang

**Affiliations:** ^1^ Department of Obstetrics and Gynecology, Chongqing Health Center for Women and Children, Women and Children’s Hospital of Chongqing Medical University, Chongqing, China; ^2^ Key Laboratory of Obstetrics & Gynecologic and Pediatric Diseases and Birth Defects of Ministry of Education, Development and Related Diseases of Women and Children Key Laboratory of Sichuan Province, West China Second University Hospital, Sichuan University, Chengdu, Sichuan, China; ^3^ Thoracic Surgery Branch, Center for Cancer Research, National Cancer Institute (NCI), Bethesda, MD, United States

**Keywords:** tumor microenvironment (TME), metabolic reprogramming, immunotherapy, metabolic pathways, immune responses, prognostic models

The tumor microenvironment (TME) is the surrounding milieu of tumor cells, including adjacent blood vessels, immune cells, fibroblasts, bone marrow-derived inflammatory cells, various signaling molecules, and extracellular matrix. This milieu plays a crucial role in the growth, proliferation, and survival of tumor cells (1). The tumor microenvironment is characterized by significant hypoxia (2), low pH levels, and a scarcity of nutrients (3). Rapid growth and significant swelling of tumor tissues, coupled with an underdeveloped vascular system within, lead to insufficient oxygen supply, giving the tumor microenvironment its overall hypoxic trait (4). Insufficient oxygen forces tumor cells to rely on anaerobic glycolysis for energy, causing lactic acid buildup and thus lowering the microenvironment’s pH. Moreover, tumor cells adjust to diverse microenvironments by modifying their metabolic pathways (termed “metabolic reprogramming”) to fulfill their unique needs for energy and perform specific functions. Metabolic reprogramming is driven collaboratively by oncogenic alterations in the cells and the influence of cytokines within the tumor microenvironment (5). Research into these metabolic pathways and their impact on immune responses within the tumor microenvironment is expected to lead to breakthrough therapies that substantially enhance patient outcomes.

In addition to established immunotherapeutic strategies, understanding the metabolic environment within tumors was critical for predicting treatment outcomes and enhancing therapy efficacy. This Research Topic collected several groundbreaking studies that explored this area. Using bioinformatics methods, Li et al. developed a hypoxia risk model for breast cancer by identifying key hypoxia-related genes such as NFIL3, SERPINE1, FOS, BGN, EGFR, and SRPX. Their model acted as an independent prognostic indicator, and highlighted the role of oxygen deprivation in cancer heterogeneity, recurrence, metastasis, and resistance. On the other hand, Zhang et al. reviewed the metabolic heterogeneity in malignant melanoma, with a specific focus on the alterations in glycolysis and oxidative phosphorylation (OXPHOS) that occurred during metabolic reprogramming. They observed that metabolic reprogramming led to hypoxia, glucose scarcity, and acidity within the tumor microenvironment (TME), conditions that collectively suppressed immune cell function and reduced the effectiveness of immunotherapy. Moreover, research indicated that proteins like LDH, MCT1/4, and PGC-1α were vital biomarkers for predicting metabolic patterns in melanoma behavior. They suggested that utilizing metabolic modulators to improve the TME could enhance the effectiveness of immunotherapy.

Exploring innovative strategies to enhance immunotherapy effectiveness, Mishchenko et al. and Wu et al. have provided comprehensive reviews on different mechanisms within the tumor microenvironment that could potentially improve treatment outcomes. Mishchenko et al. focused on the challenges of improving glioma immunotherapy, particularly the effects of inducing immunogenic cell death (ICD) to facilitate Th17 cell migration into the tumor microenvironment. By transforming it into an ‘immunologically hot’ environment, they suggested the possibility of enhancing sustained immunotherapy effectiveness. Additionally, they identified and described the primary immune characteristics of gliomas, which were thought to be crucial for predicting patient prognosis and designing personalized treatment plans. Similarly, Wu et al. summarized the latest research on the lipid metabolism of CD8+ tumor-infiltrating lymphocytes (CD8+ TILs), highlighting how fatty acids (FAs) and cholesterol may contribute to TIL dysfunction in the TME. They noted the importance of targeting CD8+ T cell lipid metabolism to promote the formation of memory phenotype cells, thereby potentially extending the duration of effective cancer immunotherapy. These studies collectively advance our understanding of how modifying the TME could impact the efficacy of immunotherapies, indication a multifaceted approach to cancer treatment that involves both metabolic and immunogenic strategies.

Recent advances in cancer research have highlighted the significant role of metabolic profiling in enhancing the effectiveness of immunotherapies. Huang et al. utilized clinical information and RNA sequencing data from the TCGA and GEO databases to build a prognostic model based on fatty acid metabolism genes in colorectal cancer. This model has shown potential in predicting how patients will respond to immunotherapy, offering a new tool to guide treatment decisions. In a similar vein, Katopodi et al. discussed the regulatory role of metabolic reprogramming in immune cells, suggesting that enhancing the immunogenicity of cancer cells could potentially expand the range of tumors that respond effectively to immunotherapy. They proposed innovative combinations of immunotherapy and metabolic interventions to maximize treatment efficacy. Further exploring metabolic influences in oncology, Tan et al. used 18F-FDG PET/CT scan data from 253 early NSCLC patients to assess how glucose metabolism reprogramming in the primary lesion and remote organs correlated with tumor size. They reported that glucose metabolism increases in the tumor and decreases in the liver as the tumor enlarges, and proposed that metabolic levels in remote organs, especially for tumors between 4-7cm, could serve as effective prognostic indicators for NSCLC. This comprehensive approach to studying metabolic changes offers a broader understanding of how these factors can be manipulated to improve patient outcomes in various cancer types.

Tumor cells manage to escape immune surveillance by reprogramming their metabolism, a mechanism that significantly alters the effectiveness of immunotherapy. big data analysis, help to identify new treatment targets, leading to novel diagnostic and therapeutic approaches for cancer treatment, and thus optimizing the design of treatment strategies. In brief, through detailed research on the metabolic diversity of immune cells within the tumor microenvironment, we can deepen our understanding of their role in combating immune resistance.

## Author contributions

JX: Writing – original draft. HW: Supervision, Writing – review & editing. XL: Supervision, Writing – review & editing.
